# Host and Microbial Factors in Regulation of T Cells in the Intestine

**DOI:** 10.3389/fimmu.2013.00141

**Published:** 2013-06-10

**Authors:** Chang H. Kim

**Affiliations:** ^1^Laboratory of Immunology and Hematopoiesis, Department of Comparative Pathobiology, Center for Cancer Research, Purdue UniversityWest Lafayette, IN, USA

**Keywords:** intestine, retinoic acid, commensal bacteria, T cells, Th17, FoxP3, dendritic cells

## Abstract

The intestine is divided into specialized tissue areas that provide distinct microenvironments for T cells. Regulation of T-cell responses in the gut has been a major focus of recent research activities in the field. T cells in the intestine are regulated by the interplay between host and microbial factors. In the small intestine, retinoic acid (RA) is a major tissue factor that plays important roles in regulation of immune responses. In the large intestine, the influence of RA diminishes, but that of commensal bacterial products increases. RA, gut microbiota, and inflammatory mediators co-regulate differentiation, distribution, and/or effector functions of T cells. Coordinated regulation of immune responses by these factors promotes well-balanced immunity and immune tolerance. Dysregulation of this process can increase infection and inflammatory diseases.

## Introduction

T cells are highly plastic and readily adopt new phenotypes in response to changes in environmental cues. T cells are heterogeneous in T-cell receptor (TCR) specificity, trafficking ability, and effector phenotype. Specialized organs and tissue areas produce regulatory factors that stimulate and change the T-cell phenotype, and only the T cells that have the capacity to respond to these tissue factors can expand and persist in specialized tissue sites. Tissue factors can be any molecules produced constitutively or inducibly in specialized tissue areas and regulate T cells. These factors include host metabolites, microbe-associated molecular patterns (MAMPs), microbial metabolites, cytokines, hormones, and inflammatory mediators. Tissue factors can promote or suppress the activation, expansion, differentiation, and survival of T cells. They can act on T cells during TCR activation and skew the phenotype of differentiating T cells. Tissue factors can also affect the phenotype of antigen-presenting cells (APC) such as dendritic cells (DCs) to indirectly affect T cells. Antigen and co-stimulation signals are primarily provided by APC for T-cell activation. However, these signals alone are not sufficient to effectively drive or skew T-cell differentiation for immune responses tailored to specific pathogens or inflammatory responses. Cytokines play central roles in T-cell differentiation. They can be produced by not only DCs but also other cells in tissues. Production of cytokines by DCs and tissue cells can be regulated by the tissue factors for indirect regulation of T cells. Together with cytokines, tissue factors play important roles in determining the fate of T cells in specialized tissue microenvironments. T-cell responses in the intestine are regulated by the interplay between host and microbial factors. Retinoic acid (RA) and gut microbiota distinctively regulate differentiation, migration, and effector functions of T cells. Moreover, inflammatory mediators such as prostaglandins can modify the effects of tissue factors. In this article, we will discuss the interacting roles of these factors in regulation of T cells in the intestine.

## Basic Factors for T-Cell Activation and Differentiation

The intestine has the highest levels of Th17, Th22, and FoxP3^+^ T cells in the body. Thus, one can say that the intestine produces more (in quantity and/or number of different types) factors that support the effector or suppressor T-cell subsets. Th1 cells produce IFN-γ; Th17 cells produce IL-17; Th22 cells produce IL-22; Th2 cells produce IL-4/IL-5/IL-13; Tr1 cells produce IL-10; regulatory T cells (Tregs) express FoxP3 and may produce IL-10, IL-35, and TGFβ1 as their effector cytokines (Zhou et al., 2009; Witte et al., 2010; Pot et al., 2011). In addition, Th9 cells produce IL-9 (Kaplan, 2013). Generation of these T-cell subsets requires distinct cytokine signals and intracellular molecules (Bluestone et al., 2009; Zhou et al., 2009).

Activation of TCRs is required for cytokine and tissue factors to affect T-cell differentiation. TCRs are activated by antigen peptides presented on MHC molecules expressed by APC. Antigens that activate TCR are the primary factor that induces T-cell activation (Sundrud and Nolan, 2010). The affinity and antigen-specific activation of TCR create various activation signals for heterogeneous outcomes (Garbi et al., 2010; Edwards and Evavold, 2011). TCR activation leads to a series of cell signaling events leading to activation of several key transcription factors such as AP-1, NFAT, and NFkB (Sundrud and Nolan, 2010). For proper activation of T cells, additional signals should come from co-stimulatory receptors such as CD28 (Acuto and Michel, 2003). Other co-stimulatory receptors include OX40, ICOS, CD137, GITR, and LIGHT for survival, expansion and differentiation of T cells (Redmond et al., 2009; Chen and Flies, 2013). Also, there are co-inhibitory receptors such as CTLA4, PD1, and BTLA to dampen or modify the TCR signaling (Sumpter and Thomson, 2011; Pasero et al., 2012). Antigen and co-stimulation signals can activate T cells but these two are not sufficient for generation of functionally specialized effector or suppressor T cells in the periphery.

For efficient activation, expansion, and differentiation of T cells, major T cell-activating and differentiating cytokines are required (Korn et al., 2009; Zhou et al., 2009; Kaplan, 2013). IL-2 is a prototype cytokine for proliferation and differentiation of T cells into Tregs and effector T cells (Liao et al., 2011). However, IL-2 suppresses the induction of Th17 cells. IL-4 is a cytokine that induces T-cell differentiation into Th2 cells. However, IL-4 inhibits the generation of induced FoxP3^+^ T cells (Nagase et al., 2007; Dardalhon et al., 2008). IL-6 and IL-23 promote the generation of Th17 cells. IL-12 promotes the generation of Th1 cells but suppresses that of Th2 cells. IL-7 and IL-15 promote homeostatic proliferation of T cells (Carrette and Surh, 2012; Hong et al., 2012). IL-21 promotes the generation of T-FH and other T cells (Crotty, 2011). IL-10 and IL-27 promote the generation of T cells producing IL-10 (Tr1) (Awasthi et al., 2007; Batten et al., 2008). These cytokines induce the activation and expression of subset-specific master transcription factors such as RORγt/STAT3 (Th17 cells), GATA3/STAT4 (Th2), T-bet/STAT6 (Th1), FoxP3/STAT5 (Tregs), and c-Maf/AHR (Tr1) (Korn et al., 2009; Zhou et al., 2009; Pot et al., 2011). The cytokines can generate specialized effector T cells but not necessarily tissue-specific T cells.

## Effector T Cells have Tissue-Specific Migratory Behaviors

The tissue-specificity of T cells is mainly regulated by trafficking receptors. T cells are arguably the most migratory immune cells in the body. The migration ability of T cells is defined by the trafficking receptors they express. Naïve T cells made in the thymus uniformly express CCR7 and CXCR4 for migration into the secondary lymphoid tissues (Campbell et al., 2003). Additionally, naïve T cells express CD62L for interaction with peripheral node addressin (PNAd) expressed on high endothelial cell venules (HEV) in the T-cell areas of lymph nodes (von Andrian, 1996). Because of their trafficking-receptor phenotype, naïve CD4^+^ T cells seldom migrate to non-lymphoid tissues. Naïve CD4^+^ T cells also express low levels of α4β7 to assure migration of some naïve T cells to gut-associated lymphoid tissues at a basal level (Mackay et al., 1996; Rott et al., 1996). T cells change their trafficking-receptor phenotype during T-cell activation (Kim et al., 2001; Kim, 2006; Lee et al., 2007). This is so called “the trafficking-receptor switch in the secondary lymphoid tissues,” and it is driven by the antigen priming process regulated by DCs (Kim et al., 2003; Lee et al., 2007). Interestingly, the outcome of the trafficking receptor switch on antigen-primed T cells is not uniform. Rather, it is heterogeneous depending on the tissue sites and conditions of antigen priming. This heterogeneity in the trafficking-receptor switch generates tissue-specific effector or suppressor T cells. Tissue factors are drained into lymph nodes together with antigens to activate T cells. A common change occurring during T-cell activation is reduced expression of CCR7 and CD62L, two of the best known homing receptors for migration into secondary lymphoid tissues. Some memory T cells retain these receptors to come back to lymphoid tissues. Memory/effector type chemokine receptors include CCR1-6, CCR8, CCR9, CCR10, CXCR3, CXCR5, and CXCR6 (Kim, 2005). In mesenteric lymph nodes (MLN) and Peyer’s patches, many T cells up-regulate CCR9 and α4β7. In peripheral lymph nodes (PLN), P-selectin glycoprotein ligand-1 (PSGL-1), CCR4, and CCR8 are expressed on T cells to make skin-homing cells (Ohmori et al., 2006). Th1 cells have the tendency to express CXCR3, CCR5, and CXCR6 (Kim et al., 2001). Th2 cells express CCR4, CCR8, and/or CRTH2 (Campbell et al., 1999; Nagata et al., 1999). Th17 cells express CCR6 and most memory/effector type chemokine receptors (Annunziato et al., 2007; Hirota et al., 2007; Lim et al., 2008). Trafficking receptors for other tissues are less clear but generally the non-gut memory/effector receptors such as P-selectin glycoprotein ligand-1 (PSGL-1), E-selectin ligand-1 (ESL-1), CXCR3, CCR5, and CCR4 are expressed highly by the T cells migrating to inflamed tissues (Kim, 2006, 2009).

Certain memory/effector T cells do not actively recirculate through the blood system but rather reside in non-lymphoid tissues such as skin and intestine (Gebhardt et al., 2009; Masopust et al., 2010; Wakim et al., 2010). These T cells are termed tissue-resident memory T cells (TRM) and play important roles in fighting pathogens and mediating tissue inflammation. TRM have been extensively studied for CD8^+^ T cells, but some CD4^+^ T cells have this phenotype as well (Gebhardt et al., 2011). TRM express CD69 and CD103 but are low in expression of CCR7 and CD62L (Hofmann and Pircher, 2011; Shinoda et al., 2012). CD69 down-regulates S1P1, which is required for recirculation of T cells (Shiow et al., 2006).

## RA is an Intestinal Tissue Factor

Retinoic acid is produced from retinol absorbed in the gut. Epithelial cells express cellular retinol binding proteins such as CRBP and CRBP II for retinoid uptake from the gut lumen (Levin, 1993). Retinol can be transported and stored in the liver and fat. Accumulation of retinol and RA at high levels in tissues is toxic, which is prevented by RA-synthesizing and degrading enzymes. Retinol is metabolized into all-trans-RA (At-RA) and 9-cis-RA (Duester, 2000; Mark et al., 2006). The conversion of retinol into RAs is regulated by several enzymes such as alcohol dehydrogenase and retinal dehydrogenases. Expression of these enzymes is tightly regulated in cells and tissues. RA functions locally and globally. As established for embryo development, RA is produced by specialized cells and affects cell proliferation and death in tissue microenvironments. ALDH1a1 and ALDH1a2, encoded respectively by *Aldh1a1* and *Aldh1a2* gene, oxidize retinal to make RA. Expression of ALDH1a2 and production of RA are induced by a number of factors including RA, PPARγ ligands (Szatmari et al., 2006; Housley et al., 2009), toll-like receptors (TLR) ligands, GM-CSF and IL-4 (Yokota et al., 2009). GM-CSF and IL-4 cooperatively induce ALDH1a2. RA is degraded by CYP26 (Haque and Anreola, 1998). CYP26 is induced by RA or MAMPs that activate primarily through TLRs to limit RA availability in tissue microenvironments and during immune responses.

Retinoic acid is present at nanomolar levels in the blood circulation (Napoli et al., 1985). Tissues such as the intestine, liver, and eyes have high expression of the RA-producing enzymes (Niederreither et al., 2002), and the RA level is expected to be high in these tissues. In the intestine, epithelial cells, DCs, and macrophages express RA-synthesizing enzymes and produce RA. DCs and macrophages express ALDH1a1 and ALDH1a2 and can present RA for T cells undergoing activation (Iwata and Yokota, 2011). The RA produced by intestinal epithelial cells would effectively affect the T cells in the intraepithelial compartment.

## RA Regulates T-Cell Effector Function

Retinoic acid signals through RAR and RXR heterodimers. T cells highly express RARα and RXRs (Iwata et al., 2003; Kang et al., 2007). Expression of RARα in T cells is augmented by RA (Halevy et al., 1994; Kang et al., 2007). The first function of RA reported for T cells is enhancement of cytotoxic T-cell function against allogeneic tumor cells (Dennert and Lotan, 1978; Dennert et al., 1979). RA is considered an anti-cancer agent for its activity to decrease tumor growth (Tang and Gudas, 2011). The relative contributions of the RA effect on tumor cells versus immune cells have yet to be determined. While RA promotes IL-2 and IL-2Rβ expression by T cells (Dennert, 1985; Ballow et al., 1997; Sidell et al., 1997), the positive effect of RA on anti-tumor immunity is at odds with the anti-inflammatory effect of RA and related RA analogs (retinoids) in the immune system (Newton et al., 1986). Retinoids ameliorate cutaneous inflammation caused by acne or lupus erythematosus. RA therapies decreased T-cell numbers in inflamed skin lesions. Others reported that T cells were even increased after treatment with RA in normal skin (Fisher et al., 1991). It seems that RA would decrease inflammatory T cells but may be required for maintaining some T cells in the skin in the steady state. The positive effect of RA on T cells is perhaps due to its cofactor function in T-cell activation. T cells did not proliferate properly in the absence of retinol or its metabolites (Garbe et al., 1992). The negative effect is perhaps mediated through induction of a Treg phenotype in T cells or direct suppression of effector T cells (Stosic-Grujicic and Ejdus, 1994).

**Figure 1:**
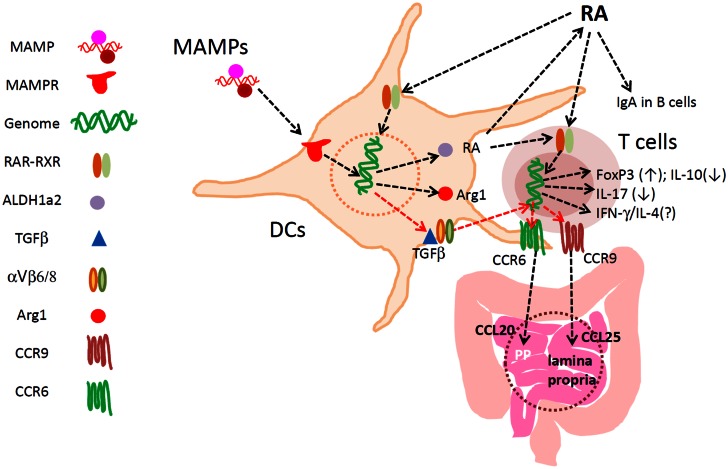
**Major functions of RA in regulation of DCs and T cells in the intestine**. RA develops specialized DCs with the capacity to express ALDH1a2, TGFβ1, Arg1, and other regulators of lymphocytes. RA directly activates T cells for induction of CCR9 and Itgα4, the latter of which pairs with Itg-β7 to make Itg α4β7. These gut-homing receptors are required for T and B cell migration to the lamina propria of the small intestine. α4β7 is important also for lymphocyte migration into the colon, Peyer’s patches, and mesenteric lymph node. Up-regulated ALDH1a2 increases RA production from retinaldehyde. Biologically active TGFβ1 is also produced by αVβ6/β8-expressing mucosal DCs to induce FoxP3^+^ T cells in the gut-associated lymphoid tissues. TGFβ1 induces CCR6, whereas RA induces CCR9 and Itg-α4. Arginase1 is induced by RA in DCs to deplete available arginine, and this promotes the generation of FoxP3^+^ T cells. RA at physiological concentrations does not decrease the numbers of Th17 cells and other effector T cells. Rather, through induction of the gut-homing receptors, RA is required for normal population of major T-cell subsets and IgA^+^ B cells in the intestine. RA, while it induces FoxP3^+^ T cells, suppresses the formation of IL-10^+^ T cells, leading to enrichment of FoxP3^+^ T cells at the expense of IL-10^+^ T cells. Thus, RA helps create a unique blend of effector and regulatory T cells effective in protection of the small intestine. In the colon, the influence of microbial factors increases to either promote or suppress RA-regulated T cells and DCs.

Another potential function of RA in regulation of T cells is their effect on Th1/2 polarization. In vitamin A-deficient mice, Th1 cells were increased at the expense of Th2 cells (Cantorna et al., 1995). The low Th2 response is in line with the function of RA in enhancing Th2 cells, a process mediated by the RXR pathway (Hoag et al., 2002; Stephensen et al., 2002; Iwata et al., 2003). In another study, Th2 and Tr1 cells were increased and Th1 cells were somewhat decreased in vitamin A deficiency (Stephensen et al., 2004). Our study, published in 2009 (Kang et al., 2009), showed that neither hypo- nor hyper-vitamin A condition had significant changes in Th1 and Th2 cells. The only exception was the small intestine, where all effector T cells including Th1 and Th2 cells were decreased. In the “pinkie” mice where RXR function is insufficient due to a mutation, the Th1 response was greatly increased (Du et al., 2005). This effect of the mutation, however, is not entirely due to RA signaling deficiency as RXRs pair also with other nuclear hormone receptors such as vitamin D receptor (VDR), peroxisome proliferator-activated receptors (PPARs), liver X receptor (LXR), bile acid/farnesoid X receptor (FXR), androstane receptor (CAR), pregnane X receptor (PXR), and thyroid hormone receptor (TR). Overall, vitamin A or RA can affect Th1 and Th2 responses. While the mechanism for this regulation is still unclear, it would be a mixture of direct and indirect regulation. RA can support T-cell activation for basic effector functions. However, RA does not act as a Th1/2 cell polarizing agent. RA can act on other cells, such as DCs and Tregs, to indirectly regulate the Th1/Th2 response. Important functions of RA in regulation of T cells and DCs are highlighted in Figure 1.

**Figure 2:**
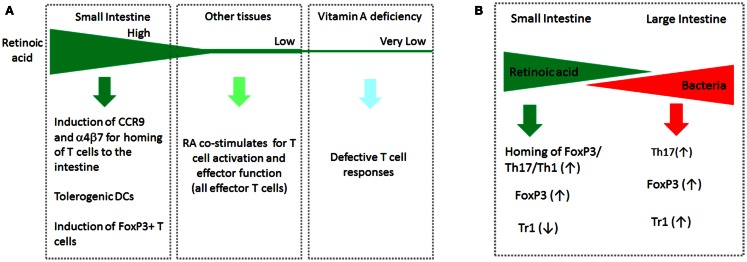
**The gradients of RA and bacterial products in the intestinal tract and systemic tissue sites cooperatively regulate T cells**. **(A)** Induction of CCR9 by RA occurs at high RA concentrations. The function of RA to support the basic effector T-cell response occurs at relatively low RA concentrations. In vitamin A deficiency, the basic T-cell response such as generation and migration of effector T cells is impaired. **(B)** While RA boosts the generation of FoxP3^+^ T cells and homing of all T-cell subsets to the small intestine, it suppresses the formation of IL-10-producing Tr1 cells. In the large intestine, the RA concentration decreases, whereas commensal bacterial cells increase in number. In this tissue environment, it is the microbial products and TLR-mediated signals that are dominant in regulation of T cells. Bacteria-derived MAMPs affect many types of T cells in a microbe-specific manner. This segment-dependent distribution of the two classes of regulatory factors would help mount immune responses necessary for different segments of the intestine.

Interestingly, the functions of RA at low and high concentrations appear different from each other. RA at high concentrations (>5 nM) induces tolerogenic APC and FoxP3^+^ T cells (and gut-homing receptors as discussed later in this article). RA at low concentrations (<3 nM) is required for optimal activation of T cells for formation of effector T cells. High concentrations of RA are found in the small intestine and possibly in other tissues where ALDH1a1 and ALDH1a2 are highly expressed. Most tissues, however, would have low concentrations of RA, which are just enough to support the general effector T-cell response but not the homing receptor expression. In this regard, it has been observed that the RA signal is required to mount an effector T-cell response during infection (Hall et al., 2011). In vitamin A or RARα deficiency, effector T cells fail to migrate and perform their functions in tissue sites of active immune responses. This phenomenon has been observed in infection and graft rejection models (Hall et al., 2011; Pino-Lagos et al., 2011). Paradoxically, RARα agonists have been used to suppress graft rejection responses (Seino et al., 2004). Again, the impact of RA is determined, in part, by the available concentrations of RA in the body. This point is summarized in Figure 2A.

## Induction of Gut-Homing Receptors by RA Generates the T Cells Populating the Intestine

A clear function of RA is to induce gut-homing receptors and to suppress lymph node/non-gut tissue-homing receptors on T cells. RA directly induces CCR9 and α4β7 and decreases CCR7 and CD62L expression (Iwata et al., 2004; Kang et al., 2007). RAR activation is important for the induction of gut-homing receptors, and co-activation of RXRs further boosts the effect of RAR activation (Takeuchi et al., 2010). α4β7 is composed of Itg-α4 and Itg-β7 subunits. Itg-α4 is induced by RA in T cells (Kang et al., 2011). Itg-β7 is constitutively induced during T-cell activation and further induced by RA and TGFβ1 (Lim et al., 1998; Kang et al., 2011). CCR9 is the small intestine-specific receptor, and α4β7 is a pan-gut-homing receptor (Kim, 2005). CCL25 is the chemokine that activates CCR9 and attracts CCR9-expressing T cells (Kunkel et al., 2000; Wurbel et al., 2000). Induction of the two gut-homing receptors by RA occurs in T cells (both CD4 and CD8) and B cells (Iwata et al., 2004; Saurer et al., 2007; Svensson et al., 2008; Mora and von Andrian, 2009). RA-dependent expression of CCR9 and α4β7 is important for migration of all T cells, including Th1, Th2, Th17, and FoxP3^+^ T cells, into the small intestine (Kang et al., 2009; Wang et al., 2010, 2013). RA also promotes the formation of CCR9^+^ plasmacytoid DCs and α4β7^+^ pre-mucosal DCs in the bone marrow (Zeng et al., 2012). In vitamin A deficiency, T cells fail to express CCR9 and express α4β7 at reduced levels (Iwata et al., 2004; Kang et al., 2009). Signaling through RARα and BATF are critical to normally express CCR9 and α4β7 on T cells (Wang et al., 2013). BATF, an AP-1 family transcription factor, is induced upon T-cell activation (Dorsey et al., 1995). Without BATF, induction of CCR9 and α4β7 by T cells did not occur *in vitro* and *in vivo* (Wang et al., 2013). This led to defective migration and function of effector T cells in the intestine. BATF-deficient Tregs were also defective in migration and suppression of T-cell mediated tissue inflammation in the intestine (Wang et al., 2013). Oral immunization induces Tregs in the intestine for promotion of immune tolerance to the antigens (Chen et al., 2003). CCR9- or vitamin A-deficient mice fail to induce oral immune tolerance because T cells, including Tregs, cannot migrate to the small intestine (Strober, 2008; Cassani et al., 2011).

## RA Induces FoxP3^+^ T Cells for Promotion of Immune Tolerance

Retinoic acid promotes the formation of FoxP3^+^ T cells from naïve T cells (Benson et al., 2007; Coombes et al., 2007; Kang et al., 2007; Mucida and Cheroutre, 2007; Mucida et al., 2007; Schambach et al., 2007; Sun et al., 2007; Elias et al., 2008). RA can induce FoxP3 expression when added during activation of naïve CD4^+^ T cells. This process is enhanced by TGFβ1. It is still not completely understood how RA induces FoxP3 gene expression. Direct and indirect roles have been proposed. Expression of the FoxP3 gene is induced by a number of different ways (Maruyama et al., 2011). RA can promote some of these pathways to induce the FoxP3 gene. An indirect mechanism through suppression of cytokines produced from effector T cells was proposed (Hill et al., 2008). In this regard, IL-4 and IL-6 can suppress the induction of FoxP3 expression (Kastner et al., 2010). RA suppresses the expression of these cytokines to indirectly induce the expression of FoxP3 gene by TGFβ1. Similarly, RA can affect the expression of key transcription factors such as STAT6, which regulates IL-4 expression. RA suppresses the function of STAT6 in an unknown manner to support FoxP3 induction by TGFβ1 (Takaki et al., 2008). The RA function in induction of FoxP3 expression is not through regulation of STAT3/STAT5 (Elias et al., 2008), which is activated by many cytokines and growth factors including IL-2. IL-2 is required for normal induction of FoxP3^+^ T cells in the periphery (Cheng et al., 2011).

While RA enhances FoxP3^+^ T cells, RA suppresses another major subset of Tregs, IL-10-producing T cells (Tr1 cells) (Maynard et al., 2009). In vitamin A deficiency, IL-10^+^ T-cell numbers were increased. Thus, the suppressive function of RA on IL-10^+^ T cells has been verified *in vivo*. The RA acting on T cells is thought to be derived from DCs and affects T cells in a paracrine manner. DCs highly express ALDH1a2 to produce RA (Iwata, 2009). Defective generation of tolerogenic DCs in the intestine provides another explanation for the dysregulated T-cell response in vitamin A deficiency. The effect of RA on DCs is discussed in detail in the next section.

## RA Affects the Phenotype of DCs and Macrophages

While RA can act directly on T cells undergoing activation, it can also change the phenotype of DCs and macrophages to indirectly affect T-cell differentiation and function. RA regulates the differentiation of myeloid cells (Breitman et al., 1980; Takenaga et al., 1980; Flynn et al., 1983). High retinol conditions promote the differentiation of mouse hematopoietic progenitor cells into DCs, whereas low retinol conditions generate more neutrophils *in vitro* (Hengesbach and Hoag, 2004). In general, RA generates DCs with a low T-cell activation capacity. It was observed that RA can generate DCs from human cord blood monocytes with a decreased ability to produce IL-12 and activate T cells (Tao et al., 2006). Similarly, 9-cis-RA, which activates both RAR and RXR, induced IL-10 but decreased IL-12 expression in cultured human monocytes. It was also observed that 9-cis-RA can interfere with human DC maturation (Zapata-Gonzalez et al., 2007). RA also drives bioactive TGFβ production by DCs through suppression of SOCS3 and subsequent activation of STAT3 (Feng et al., 2010). RA decreased the adherence of BM-DCs and induced expression of matrix metalloproteinase-9 (Lackey et al., 2008). The function of RA in generating tolerogenic DCs is in line with their ability to induce FoxP3^+^ Tregs (Feng et al., 2010). RA induces expression of Arginase1, which enhances the FoxP3^+^ T cell-inducing ability of DCs (Chang et al., 2013). The mouse *Arginase1* gene promoter has RAR binding sites which mediate the expression of *Arginase1* in response to RA. RA-treated DCs were not able to induce Th17 cells, whereas DCs developed with an RAR antagonist were highly efficient in induction of Th17 cells (Chang et al., 2013). Thus, RA generates DCs with a reduced ability to make effector T cells such as Th17 cells but an enhanced ability to induce FoxP3^+^ T cells. The function of RA in regulation of DCs and T cells in the intestine is summarized in Figure 1.

On the contrary, RA can also induce inflammatory DCs in certain conditions. It has been observed that RA can promote differentiation of human monocytes into IL-12-producing DCs that express CD1a (Mohty et al., 2003). In another study, monocyte-derived DCs that were pretreated with RA, acquired the ability to secrete IL-6 and TGFβ1 (Saurer et al., 2007). RA, together with IL-15, can activate DCs to produce IL-12 (DePaolo et al., 2011). This process is mediated by JNK activation and may induce an inflammatory T-cell response to dietary antigens. Thus, while RA promotes tolerogenic DCs in a steady state, it can also generate proinflammatory DCs in response to other factors.

RA changes the phenotype of DCs into intestine-residing DCs. Small intestine lamina propria-residing DCs can produce RA and efficiently induce FoxP3^+^ T cells (Sun et al., 2007). Certain subsets of DCs and macrophages highly express ALDH1a2 and produce RA. These cells are efficient in induction of FoxP3^+^ T cells. Another factor is TGFβ1, which is expressed as the inactive latent form. CD103^+^ DCs in the intestine express αV-containing integrins, which binds through the RGD motif of LAP-TGFβ and activates the cytokine for induction of FoxP3^+^ T cells (Worthington et al., 2011). However, the RA-producing and FoxP3-inducing ability is not unique to the mucosal DCs. In skin-draining lymph nodes, DCs that don’t express CD103 can produce RA and induce FoxP3^+^ T cells (Guilliams et al., 2010). A limiting factor in this case is the availability of retinol in the skin. Compared to the gut where retinol is absorbed, the retinol level in the skin is expected to be low. In vitamin A deficiency, the DCs in gut-associated lymphoid tissues and lamina propria abnormally expressed langerin, which is typically expressed by skin-residing dermal langerin^+^ DCs (Chang et al., 2010). The DCs in the spleen of vitamin A-depleted animals were also abnormal with increased CD8α^+^ lymphoid DCs (Duriancik and Hoag, 2010).

Another function of mucosal DCs is to induce IgA-producing B cells through their RA-producing ability. RA promotes IgA production in B cells (Tokuyama and Tokuyama, 1995). DCs in the MLN and Peyer’s patches have the capacity to induce IgA-producing B cells in co-culture (Mora et al., 2006), a process enhanced by IgA-inducing cytokines such as IL-5 and IL-6. Human monocyte-derived DCs, developed in the presence of RA *in vitro*, had a similar ability to induce IgA-producing B cells (Saurer et al., 2007). The DC-induced class switch recombination can occur in any lymphoid tissues but it is most clear in isolated lymphoid follicles where no or few T cells are present (Tsuji et al., 2008). In addition to regular myeloid DCs, follicular DCs (FDCs) are present in germinal centers and regulate B cell differentiation into plasma B cells. Stimulation of FDCs by bacterial products and RA synergistically enhanced the ability of FDCs to induce IgA^+^ B cells (Suzuki et al., 2010). This process is mediated by enhanced expression of the chemokine CXCL13, the survival factor BAFF, and TGFβ1.

## Commensal Bacteria Produce Tissue Factors That Affect T Cells

Commensal bacteria are present mainly in the large intestine (Figure 2B). They are also found in low numbers in the small intestine, particularly in the ileum. Commensal bacteria significantly affect immune responses to pathogens and are themselves a subject of immune responses during infection by pathogens (Hand et al., 2012). Successful population of T cells in the intestinal tissue requires host-specific microbiota (Chung et al., 2012). In other words, the human microbiota is not effective in activating T cells in mice, and it is expected that the same is true for the mouse microbiota in humans. This information suggests that the microbiota that grows in a species has been selected by the immunological and other pressures of the host. Alternatively, the host and microbiota may have been co-evolved over a long time period to support and regulate each other.

Comparison of germ-free (GF) and specific pathogen-free (SPF) mice revealed the important role of the gut microbiota in normal population of the intestine with T cells. A GF condition did not affect the number of FoxP3^+^ T cells in the small intestine but it greatly decreased the T-cell population in the colon (Atarashi et al., 2011). This resistance of the small intestinal T cells is interesting but the reason for this resistance is unknown. A GF condition decreased the presence of Th17 cells in both the small and large intestine. Th1 cells in the intestine were not affected, but IL-10-producing T cells were decreased in GF mice (Atarashi et al., 2011). TLRs play important roles in conveying some of the microbial signals to host cells for regulation of T cells. The important roles of TLR-activating molecules in regulating innate immune cells are well established (Mills, 2011). T cells express certain TLRs and can be under the direct control of microbes. For example, TLR2 signaling in T cells enhances the generation of Th17 cells (Reynolds et al., 2010).

It was observed more than a decade ago utilizing monoassociating GF mice that segmented filamentous bacteria (SFB) potently stimulated the mucosal immune system (Talham et al., 1999). SFB increased activated T cells and IgA production in both the small and large intestine. In contrast, the effects of *Clostridia* bacteria on T cells and B cells were largely limited to the large intestine (Talham et al., 1999). SFB, while inefficient in inducing T-cell-mediated colitis by themselves, were required to induce colitis together with a defined cocktail of SPF bacteria (Stepankova et al., 2007). SFB were required for efficient formation of T cells expressing IL-17 and IL-22 in the intestine (Ivanov et al., 2009). These effector T cells confer enhanced immunity against *C. rodentium* infection. SFB are physically associated with intestinal epithelial cells. The intestinal epithelial cell surfaces that SFB interact with are devoid of microvilli for tight interaction between SFB and epithelial cells. This interaction would, in part, make SFB highly effective in stimulating the mucosal immune system. Intestinal colonization with SFB changes the gene expression profile of the host cells in the intestine. Serum amyloid A (SAA), induced by SFB in the terminal ileum, can stimulate DCs to promote Th17 cell differentiation (Ivanov et al., 2009). SFB also affect T-cell responses in other tissues. Induction of encephalomyelitis was mediated by Th17 cells and was enhanced by SFB in the intestine (Lee et al., 2011). While GF animals didn’t develop the disease, the GF mice colonized with SFB developed encephalomyelitis.

Clusters IV and XIVa of the genus *Clostridium* are effective in inducing FoxP3^+^ T cells in the colon (Atarashi et al., 2011). Inoculation of mice with *Clostridia* decreased colitis and IgE-mediated allergic response. The determinant of *Clostridia* inducing FoxP3^+^ T cells is not clear but the effect is MyD88-dependent, suggesting potential roles of TLRs. A bacterial polysaccharide from *Bacteroides fragilis* can also increase FoxP3^+^ T cells that produce IL-10 (Mazmanian et al., 2005). TLR2 is required for induction of FoxP3 and IL-10 in T cells by the polysaccharide. This polysaccharide, when administered via oral gavage was effective in inducing Tregs and ameliorating TNBS-induced colitis in mice. The bacteroid polysaccharide decreased numbers of Th17 cells in the mesenteric lymph node and increased Th1 cells in the spleen (Round and Mazmanian, 2010). The TLR2 expressed on FoxP3^+^ T cells is important for this process, and, interestingly, colonization of *B. fragilis* in the gut was inhibited by Th17 cells in the host (Round et al., 2011). Thus, the interaction is bi-directional between the host and microbiota, resulting in sustained immune tolerance and control of commensal bacteria.

Probacteria, such as *Bifidobacteria* and *Lactobacilli*, suppress some inflammatory diseases. These probiotic bacteria can promote production of IL-10 and Tregs and ameliorate TNBS-induced colitis (Di Giacinto et al., 2005). Similarly, *Bifidobacteria* and *Lactobacilli* had protective effects on allergic responses in lungs and intestine (Lyons et al., 2010). *Bifidobacterium breve* induced IL-10-producing T cells in the large intestine and can suppress T-cell-mediated colitis (Jeon et al., 2012). DCs produce Tr1-inducing cytokines such as IL-27p28, Ebi3, and IL-10 in response to *B. breve*. The identity of the molecule(s) of *B. breve* inducing the response is unclear but this response was again dependent on Myd88 and TLR2 expressed by DCs.

Along with TLR ligands, microbial metabolites have the potential to affect tissue cells and T cells. Short chain fatty acids (SCFAs) are the most abundant microbial metabolites in the intestine. SCFAs are anaerobic fermentation products derived from dietary fibers by commensal bacteria. SCFAs constitutively activate epithelial cells in the intestine via GPR41 and GPR43 (Kim et al., 2013). This activation is important for prompt activation of epithelial cells for production of inflammatory cytokines and chemokines for mounting immune responses to pathogens, including leukocyte recruitment and induction of effector T cells (Kim et al., 2013).

## Interaction among RA, Microbial Factors, and Inflammatory Mediators

Host tissue factors such as RA and microbial factors have profound effects on T cells. The functions, and production sites and conditions of these factors are different. Therefore, these factors either positively or negatively regulate the distribution and function of T cells in the intestine. Some of the function of RA is counter-regulated by TLR activation and inflammatory signals (Maynard et al., 2009). MyD88 is a signaling molecule that mediates the signals from most TLRs except TLR3 and activates NFkB in immune cells (Feng et al., 2010). MyD88 signaling is required for optimal RA-induced expression of ALDH1a2 in DCs. The ability of mucosal DCs to induce IgA-producing B cells is partially dependent on the presence of intestinal commensal bacteria, and can be induced by LPS in culture (Massacand et al., 2008). Consistently, the TLR1/2 signaling pathway mediated by MyD88 is required for the RA-dependent function of DCs in promoting IgA-producing B cells (Wang et al., 2011). Thus, microbial factors cooperate with RA in regulation of the immune system. RA and microbial factors cross-regulate the generation of FoxP3^+^ cells and IL-10^+^ T cells. Microbial factors promote IL-10^+^ T cells but RA promotes FoxP3^+^ cells. The significance and impact of this differential regulation are yet to be established.

Prostaglandin E2 (PGE2) is produced during a variety of inflammatory responses and regulates a number of physiological and immunological processes in the body (Kalinski, 2012; Sreeramkumar et al., 2012). It has been reported that prostaglandin E2 (PGE2) is a negative regulator of ALDH1a2 through enhancing the expression of inducible cyclic AMP early repressor (ICER) (Stock et al., 2011). Blocking of PGE2 signaling greatly enhanced the RA effect on induction of ALDH1a2-producing DCs and CCR9^+^ T cells in mice (Stock et al., 2011). Thus, inflammatory mediators can reverse the RA effect on T cells. The cytokine IL-15, produced during immune responses and tissue inflammation (Perera, 2000), can also turn the tolerogenic activity of RA into inflammatory activity for DCs (Arranz and Garrote, 2011; DePaolo et al., 2011).

## Conclusion and Future Studies

In sum, host factors exemplified by RA and a myriad of microbial factors regulate each other’s activities in affecting T cells in the intestine. It is expected that inflammatory mediators further regulate the effects of RA and microbial factors. Despite the progress made so far, the functions of RA in the regulation of T cells and APC are not fully understood. The function of RA in regulation of T cells appears comprehensive and complex. While the overall positive effect of vitamin A on T-cell-mediated immunity and immune tolerance *in vivo* is established, the specific functions of RA on individual cell types including various T-cell subsets, APC, and tissue cells are yet to be elucidated. Another poorly understood area is the gene expression program regulated by RA in T cells and APC. We don’t fully comprehend how the RA signal regulates the expression of functionally important genes in T cells and APCs. RA and other signaling pathways such as TCR and co-stimulatory signals can cross-talk. This potentially important interaction warrants more studies. Interaction or cooperation between RARs and other transcription factors (e.g., BATF) is also important. Beyond RA, MAMP-regulated T-cell responses also need more studies in terms of gene expression and signaling interaction with RA or other tissue factors. These intricate interactions would be important for precise regulation of T cells in time and space to maximally benefit the host. Imbalance or dysregulation of the factors would lead to insufficient or uncontrolled T-cell activities and diseases in the intestine and other parts of the body. More studies on CD8 T cells, γδ T cells, and other functionally important T-cell subsets are needed to understand regulation of the entire T-cell network in the intestine. We are only beginning to unravel the regulatory mechanisms for these factors. It is expected that we will witness more of these host and microbial factors that can regulate T cells and APC in the future.

## Conflict of Interest Statement

The authors declare that the research was conducted in the absence of any commercial or financial relationships that could be construed as a potential conflict of interest.

## Abbreviations

RA, retinoic acid; DCs, dendritic cells; MAMPs, microbe-associated molecular patterns; PGE2, prostaglandin E2; GF, germ free; Tregs, regulatory T cells.
